# Association of Total, Added, and Natural Phosphorus Intakes with Biomarkers of Health Status and Mortality in Healthy Adults in the United States

**DOI:** 10.3390/nu14091738

**Published:** 2022-04-22

**Authors:** Kristin Fulgoni, Victor L. Fulgoni, Taylor C. Wallace

**Affiliations:** 1 Nutrition Impact, LLC, Battle Creek, MI 49014, USA; fulgonik@gmail.com; 2 Think Healthy Group, LLC, Washington, DC 20001, USA; taylor.wallace@me.com; 3 Department of Nutrition and Food Studies, George Mason University, Fairfax, VA 22030, USA

**Keywords:** phosphorus, phosphates, cardiovascular diseases, kidney, diet, risk factors, food additives

## Abstract

The Western diet is high in dietary phosphorus, partially due to added phosphorus, (i.e., phosphates) predominantly present in processed food products. Elevated serum phosphate levels, otherwise known as hyperphosphatemia, have been associated with changes in health status, of note detrimental effects on cardiovascular and renal health. However, the extent to which highly absorbed added phosphorus contributes to these changes is relatively unknown, due to its poor characterization among food composition databases. Industry-provided data on phosphorus source ingredients and ranges of added phosphorus present in food categories to enable a more accurate estimation of the total, added, and natural phosphorus intakes in the U.S. population. Using regression analyses, we then assessed relationships between estimated total, added, and natural phosphorus intakes on biomarkers of health status and mortality in individuals enrolled in the National Health and Nutrition Examination Survey (NHANES) 1988–1994 and 2001–2016 datasets. Total, added, and natural phosphorus intakes were associated with several biomarkers of health status. Added phosphorus intake was consistently inversely associated with HDL cholesterol in both men and women, whereas naturally occurring phosphorus intake was inversely correlated with the risk of elevated blood pressure. However, in most cases, the predicted impact of increases in phosphorus intake would result in small percentage changes in biomarkers. No meaningful associations between phosphorus and mortality were found, but indications of a correlation between mortality with quintiles of naturally occurring phosphorus were present, depending on covariate sets used. The disparate results for natural and added phosphorus intakes within the current study provide increased support for updating current food composition databases to more accurately account for dietary phosphorus intake as total, naturally occurring, and added phosphorus.

## 1. Introduction

Phosphorus is an essential nutrient involved in many physiological processes not limited to energy production, metabolic reactions, and cell structure. Published by the National Academy of Medicine (NAM) in 1997, the current Recommended Dietary Allowance (RDA) for phosphorus is 700 mg/d for adults 19+ years [1], yet numerous studies have shown intakes to be in excess of the RDA [2,3,4]. However, the proportion of Americans that have been reported to exceed the defined Tolerable Upper Intake Level (UL) of 4000 and 3000 mg/d for those 19–70 and 71+, respectively, is small [1,5]. Although the Dietary Reference Intake (DRI) values for phosphorus were published 25 years ago, the European Food Safety Authority (EFSA) recently reported an acceptable daily intake (ADI) for phosphates expressed as phosphorus of 40 mg/kg body weight per day, which for a 70 kg person equates to 2800 mg/d [6].

Systemic phosphate homeostasis is maintained primarily through urinary excretion [7]. The National Kidney Foundation guidelines recommend targeting serum phosphate concentrations of 2.7–4.6 mg/dL in patients with stages 3 and 4 chronic kidney disease (CKD) and 3.5–5.5 mg/dL in patients with stage 5 CKD or those receiving dialysis [8]. Phosphate binders are the only U.S. Food and Drug Administration (FDA)-approved treatment for hyperphosphatemia and are prescribed to ~80% of patients receiving dialysis [9,10]. Inadequate phosphorus excretion can lead to hyperphosphatemia or elevated serum phosphate levels [11], which has been associated with an increased number of cardiovascular and fracture hospitalizations, vascular calcification, progression of CKD, death resulting from coronary artery disease, and/or mortality in patients with CKD or those receiving hemodialysis [12,13,14,15,16,17]. While hyperphosphatemia has been a known health risk for CKD patients, recent studies suggest healthy individuals may also be at risk. Chang and colleagues showed dietary phosphorus intake greater than 1400 mg/d in patients without diabetes, cancer, CKD, or cardiovascular disease (CVD) was associated with a higher risk of all-cause mortality [2]. Higher serum phosphorus levels in healthy, young adults, were reported to be associated with higher coronary artery calcification with every 0.5 mg/dL increase in serum phosphate resulting in a 17% increased risk [18]. Similarly, in patients free of CKD and CVD at baseline, a 1 mg/dL increase in serum phosphate was associated with a 31% higher risk of CVD [19]. These recent findings, among others, have prompted more research into the health impact of added vs. natural phosphorus since inorganic added phosphorus is known to be highly absorbed by the body. Approximately 70–100% of inorganic phosphate salts are absorbed by the body in comparison to 20–50% of natural phosphorus from plant-derived foods and 40–60% from animal-derived foods [20].

Phosphate additives display a wide range of utility and versatility in processed food products by acting as effective processing aids, leavening and anti-caking agents, acidulants, emulsifiers, and stabilizers, among other uses [21]. Moore et al. found that 22.9% of food servings consumed contained inorganic added phosphorus in the form of phosphate additives [22]. Two previous studies showed that phosphate additives contribute 67–70 mg of additional phosphorus per 100 g of food to the diet [23,24]. A 2015 simulation study showed a high additive/processed food diet contributed 606 ± 125 mg more phosphorus than a low additive/processed food diet [25].

Our previous work utilizing industry-provided data on phosphorus source ingredients and ranges of added phosphorus present in food categories showed that both total and natural phosphorus intakes slightly increased between NHANES 1988–1994 and 2015–2016 data cycles, whereas added phosphorus intakes slightly decreased during this time frame. Added phosphorus currently accounts for ~11.6% of total phosphorus intake among U.S. adults 19+ years [26]. Therefore, the objective of this research was to utilize these more accurate estimates to determine if associations between total, added, and natural phosphorus intake exist among biomarkers of health status and overall mortality risk across the U.S. population.

## 2. Materials and Methods

The NHANES is a cross-sectional nationally representative survey of non-institutionalized citizens in the United States administered annually by the Centers for Disease Control and Prevention (CDC). The survey includes a dietary interview, What We Eat in America (WWEIA), combined with physical and laboratory examinations. Data for adults 19+ y from 1988–1994 through 2001–2016 NHANES surveys were utilized in the current study (the former data set has the majority of the mortality data while the latter data set has more recent intakes and physiological measures). Standard methods and study designs utilized by NHANES have been previously described [27,28]. All participants provided written informed content. NHANES protocols were approved by the National Center for Health Services (NCHS) Research Ethics Board [29].

The approach used to estimate added phosphorus in food categories was the same as in our previous work [26]. Briefly, information was collected on phosphorus source ingredients and the range of levels present in food categories from phosphate ingredient manufacturers. The minimum and maximum levels of PO4/P2O5 were multiplied by their respective molecular weights to obtain the phosphorus content by weight, which was then averaged for each food category. These values were then multiplied by the percentage of products in a food category with phosphorus ingredients as determined by an analysis of Innova Market Insights database that contains information on ingredients in foods, to obtain the average percentage of added phosphorus in the food category. Natural phosphorus was determined by subtracting the calculated added phosphorus from the total phosphorus content provided in NHANES dietary intake files. Total, natural, and added phosphorus intakes were determined by multiplying the percentage of respective phosphorus in the food item by the intake of the food.

Dietary recalls were collected using the Automated Multiple-Pass Method (AMPM) [30] and used to determine the intake of total, added, and natural phosphorus from all foods and beverages, not including dietary supplements. The first recall was collected in person while the second recall was collected via the telephone. Participants with incomplete dietary information and those who were pregnant and/or lactating were excluded from these analyses. Individual usual intakes (an estimate of longer-term intakes) of total, added, and natural phosphorus were determined using the National Cancer Institute (NCI) method [31]. Study participant characteristics were described based on total phosphorus intake.

The following laboratory measures available in NHANES were used in these analyses: serum phosphorus, systolic blood pressure (BP), diastolic BP, total cholesterol, LDL-cholesterol, HDL-cholesterol, apolipoprotein B, fasting triglycerides, fasting glucose, fasting insulin, creatinine, estimated glomerular filtration rate (GFR), total femur bone mineral content, and total femur bone mineral density. Details of methods to obtain each laboratory measure are available on the NHANES website [27]. The CVD risk scores were calculated using the American College of Cardiology/American Heart Association formula which considers age, total and HDL cholesterol, systolic BP, diabetes, and current smoking status [32]. All-cause mortality data were derived from NHANES Linked Mortality files, which link subjects enrolled in NHANES to the National Death Index (NDI) through 31 December 2015. All-cause mortality data were limited to those participants in NHANES 1988–1994 data cycles [33].

Odds and hazard ratios (99% CI) were determined with regression analyses to assess associations of individual usual intake of total, added, and natural phosphorus with laboratory measures as continuous variables. The following covariates were used for the analysis: age, age^2^, gender, race/ethnicity, physical activity level (defined as vigorous, moderate, and sedentary based on responses to a questionnaire), poverty income ratio (PIR) level (defined as the ratio of household income to government-defined poverty levels separated into three groups: <1.35, 1.35 to ≤1.85, and >1.85), and total caloric intake (defined as individual usual intake as determined using the NCI method) [31]. Body mass index (BMI) was an additional covariate for fasting glucose, insulin, and triglycerides. Logistic regressions were used to assess the association of individual usual intake of total, added, and natural phosphorus with laboratory measures as risk factors based on universally accepted levels used to define disease risk: systolic BP: ≥130 mm Hg; diastolic BP: ≥80 mm Hg; total cholesterol: ≥200 mg/dL or taking anti-hyperlipidemic medications; LDL-cholesterol: ≥100 mg/dL or taking anti-hyperlipidemic medications; low HDL-cholesterol: <40 mg/dL in males and <50 mg/dL in females or taking anti-hyperlipidemic medications; fasting triglycerides: ≥150 mg/dL or taking anti-hyperlipidemic medications; fasting glucose: ≥100 mg/dL or taking insulin or other hypoglycemic agents; and fasting insulin: ≥15 µU/mL. A final analytical sample for the above set of analyses included 39,796 participants.

Logistic regression was performed to assess the association of individual usual intake of total, added, and natural phosphorus as a continuous variable with all-cause mortality across quintiles of intake as g/d and as mg/kcal. We limited the mortality analyses to those 20–80 y at baseline (*n* = 15,020). We excluded participants with various chronic diseases, (e.g., coronary heart disease, diabetes, cancer, etc.), those taking antidiabetic medication, and those with caloric intakes in the <1st and >99th percentile. This left a final analytical sample of 10,481 (Supplementary Table S1).

A multivariate Cox regression was used to estimate all-cause mortality risk. Based on covariate sets of previous studies focused on phosphorus and mortality [2,34], we built three models for our mortality analyses:

- Adjusted for age, gender, race/ethnicity, (i.e., Mexican American, Other Hispanic, Black, Other, and White), PIR as three groups (<1.35, 1.35 to ≤1.85, and >1.85) and total caloric intake;
- Adjusted for model 1 and BMI, systolic BP, smoking status, (i.e., former, current, never), physical activity level (moderate, vigorous, sedentary), LDL cholesterol, log (albumin creatinine ratio), GFR (CKD-EPI), and serum vitamin D (<16.2 ng/mL, ≥16.2 ng/mL);
- Adjusted for model 2 and Healthy Eating Index 2015 (HEI-2015) score and serum phosphorus.

Hazard ratios (HR) and lower and upper 99th percentile confidence intervals (CIs) were generated using quintile 1 as the reference. Statistical significance was set a *p* < 0.01 and all analyses were conducted using SAS 9.4 statistical software (SAS Institute, Cary, NC, USA). Sample-weighted data were used in all statistical analyses to adjust the variance for the clustered sample design of NHANES. Regression analyses were performed using the PROC SURVEYREG function while logistic regression and generation of odds ratios were performed using the PROC SURVEYLOGISTIC function. Hazard ratios were performed using the Cox proportional hazards regression model function PROC PHREG.

## 3. Results

### 3.1. Association of Individual Usual Intake of Phosphorus with Physiological Parameters

Total phosphorus (1292 ± 11 mg/d vs. 1398 ± 17 mg/d) and naturally occurring phosphorus (1113 ± 10 mg/d vs. 1243 ± 16 mg/d) increased from 1988–1994 to 2015–2016 whereas added phosphorus intake decreased (178 ± 2.9 mg/d vs. 155 ± 4.1 mg/d) in adults (19+ y) [26].

#### 3.1.1. Added Phosphorus

Added phosphorus intake was associated with an elevated overall CVD risk score (β ± SE: 0.004 ± 0.001 increase per 100 mg added phosphorus) and increased levels of both creatinine (β ± SE: 0.01 ± 0.004 mg/dL increase per 100 mg added phosphorus), and glycohemoglobin (β ± SE: 0.07 ± 0.01% increase per 100 mg added phosphorus) in gender combined analyses. In gender-specific analyses, the association persisted with glycohemoglobin in males (β ± SE: 0.08 ± 0.02%) and CVD risk score in females (β ± SE: 0.01 ± 0.002 points).

Added phosphorus was inversely associated with HDL-cholesterol levels (β ± SE: −2.12 ± 0.28 mg/dL decrease per 100 mg added phosphorus) in gender combined analyses. In the gender-specific analyses, the decrease in HDL-cholesterol was significant in both males and females (β ± SE: −1.65 ± 0.26 and −3.12 ± 0.60 mg/dL per 100 mg added phosphorus, respectively) (Table 1).

#### 3.1.2. Natural Phosphorus

Natural phosphorus was inversely associated with diastolic BP, systolic BP, CVD risk score, and total cholesterol (β ± SE: −0.25 ± 0.05 mm Hg, −0.32 ± 0.07 mm Hg, −0.001 ± 0.0003 points, and −0.79 ± 0.19 mg/dL per 100 mg natural phosphorus, respectively), and positively associated with glycohemoglobin, HDL-cholesterol, serum phosphorus, femur bone mineral content, and femur bone mineral content density (β ± SE: 0.02 ± 0.003%, 0.26 ± 0.07 mg/dL, 0.01 ± 0.003 mg/dL, 0.26 ± 0.05 g, and 0.005 ± 0.001 g/cm^2^ per 100 mg natural phosphorus, respectively), in gender combined analyses. Most of these associations remained in gender-specific analyses (Table 1).

#### 3.1.3. Total Phosphorus

Association with total phosphorus intake followed a similar pattern as that for natural phosphorus. Total phosphorus was inversely associated with diastolic BP, systolic BP, CVD risk score, and total cholesterol (β ± SE: −0.28 ± 0.05 mmHg, −0.37 ± 0.07 mmHg, −0.001 ± 0.0003 points, and −0.82 ± 0.19 mg/dL per 100 mg total phosphorus, respectively), and positively associated with glycohemoglobin, serum phosphorus, femur bone mineral content, and femur bone mineral content density (β ± SE: 0.03 ± 0.003%, 0.02 ± 0.003 mg/dL, 0.28 ± 0.05 g and 0.01 ± 0.001 g/cm^2^ per 100 mg total phosphorus, respectively), in gender combined analyses. Additionally, a similar pattern of associations was seen in gender-specific analyses (Table 1).

### 3.2. Association of Individual Usual Intake of Phosphorus with Risk Factors of Physiological Parameters

We found a 21% higher likelihood of reduced HDL-cholesterol, for 100 mg of added phosphorus in the gender combined analyses. In the gender-specific analyses, these risks were 14% and 37% among males and females, respectively (Table 2).

A 3% lower likelihood of having elevated blood pressure for 100 mg of natural phosphorus intake was shown in the gender combined analyses; a reduced odds of elevated BP was found in males (5% less likelihood) but not in females. In females, natural phosphorus was associated with a 6% lower risk of reduced HDL cholesterol and a 7% increased risk of elevated insulin per every 100 mg of natural phosphorus (Table 2).

A 3% reduction in risk of elevated blood pressure for 100 mg of total phosphorus was shown in gender combined analyses. Similar findings were found in males (5% reduced risk), but not in females. In females, a 6% higher likelihood of elevated insulin for 100 mg of total phosphorus was shown (Table 2).

### 3.3. Hazard Ratios Analyses of Individual Usual Intake of Phosphorus with All-Cause Mortality

Quintiles of total phosphorus intake ranged from <1020 to >1630 mg/d while added and natural phosphorus ranged from <140 to >230 and <871 to >1406 mg/d, respectively (Table 3). Overall, there were no significant associations between total, added, and natural phosphorus intakes and all-cause mortality (*p* > 0.01) (Table 4). Regarding odds ratios for specific quintile groups, there were no associations between total, added, and natural phosphorus intake and all-cause mortality in covariate set 1 (Table 4). Participants in the 4th quintile of natural phosphorus showed 46% lower odds of all-cause mortality. The addition of additional covariates did not impact the results. Quintiles of phosphorus intake as mg/kcal did not show any associations with all-cause mortality (Table 5).

## 4. Discussion

Total phosphorus intake was inversely associated with systolic BP, diastolic BP, and total cholesterol and positively associated with serum phosphorus, femur bone mineral content, and femur bone mineral content density with a similar pattern of associations seen in gender-specific analyses. These associations appear to be driven mostly by natural phosphorus as similar associations were found with this intake variable. The magnitude of the associations for gender combined analyses was relatively small, <2% change in mean values per ~25% change (310 and 350 mg/d in natural and total phosphorus, respectively), in natural/total phosphorus intake, except that the change in CVD risk score was about −4% and total femur bone mineral density was about 3.5%. Added phosphorus was positively associated with creatinine and glycohemoglobin levels and inversely associated with HDL-cholesterol levels, but in the gender specific analyses only the decrease in HDL-cholesterol remained significant. Again, the magnitude of the associations for gender combined analyses was relatively small, less than 2% change in mean values per ~25% change (40 mg) added phosphorus intake. Regarding risk factors, total phosphorus was inversely associated with elevated BP, primarily driven by results in males. Again, a similar pattern to that of total phosphorus was seen for natural phosphorus except that a lower risk of reduced HDL-cholesterol was found in females. On the other hand, added phosphorus was associated with an increased risk of reduced HDL cholesterol. Levels of added phosphorus intake and serum phosphate have been shown to correlate with atherosclerosis in humans and animal models, but it is not clear whether phosphate levels are an associated factor or play a causal role. Future research warrants investigation of the role that added phosphorus may play in altering HDL cholesterol levels. Regarding mortality, no significant trends were present across the various measures of phosphorus intake.

The difference in associations of added and natural phosphorus with HDL cholesterol and the risk of reduced HDL cholesterol was unexpected. HDL cholesterol has been found to be directly associated with serum phosphorus in previous studies [18,34]. However, these findings did not consider the type of phosphorus intake. Other studies reported total/HDL-C and LCL-C/HDL-C ratios were associated with serum phosphorus and higher food additive phosphorus, respectively [19,35]. This may be similar to our findings that suggest HDL-cholesterol has an inverse relationship with added phosphorus. That said, it is hard to ascertain if these associations are directly due to added phosphorus or whether it is confounded with other food components with added phosphorus. For example, natural phosphorus from dairy, especially full-fat dairy, has been reported to promote higher HDL-cholesterol levels [36]. On the other hand, three of the five top sources of added phosphorus (cakes/pies, rolls/buns, and cookies/brownies) are higher in refined carbohydrates [26], which has also been shown to be associated with lower HDL-cholesterol levels [37].

In the current study, total and natural phosphorus were inversely associated with BP. This agrees with a previous study focused on the Atherosclerosis Risk in Communities (ARIC) and Multi-ethnic Study of Atherosclerosis (MESA) data, which found high phosphorus intake was associated with lower systolic and diastolic BP studies [38], whereas others have found associations only with lower diastolic BP [39], or no significant associations [40]. While these studies did not attempt to separate intake into natural and added phosphorus, a recent study by McClure et al. separated dietary phosphorus into animal, plant, and added sources and reported a positive association between higher additive phosphorus intake and systolic and diastolic BP [41]. This is likely due to the modulating suppression behavior of phosphorus and calcium on parathyroid hormone [42]. Decreased levels of parathyroid hormone are known to increase blood pressure [43], but it is possible this effect is due to phosphorus additives compared to natural or total phosphorus.

No associations were found between GFR and phosphorus intake in the current study. On the other hand, an association between added phosphorus and creatinine, a measure of kidney function impairment, showed creatinine levels increased slightly (0.01 ± 0.004 mg/dL) per 100 mg added phosphorus intake. According to Mayo Clinic, the typical range for serum creatinine is 0.74–1.35 mg/dL for men and 0.59–1.04 mg/dL for women [44]. An increase of 0.01 mg/dL resulting from the consumption of 100 mg of added phosphorus represents a 0.74 and 1% increase for men and women, respectively.

While we did not show a significant (*p* < 0.01) trend across phosphorus intake with all-cause mortality, others have found correlations between high total phosphorus intake (>1400 mg/d which is similar to our quintile 4 and 5 intakes) and all-cause mortality [2]. Our results suggest that this finding may be attributable to high natural phosphorus rather than added phosphorus. Previous studies have reported an association between serum phosphorus and mortality or relative risk of death [14,16,34], but it is difficult to assess the relevance of these studies to our work focusing on dietary intake due to inconsistent correlations between the two measures [4]. The covariate sets utilized mirrored those used in previous studies [2,34] with further analyses performed with more typical covariate sets for intake studies resulting in no change in results (data not shown). For our mortality analyses, we only had about 1400 deaths for the approximate 10,500 participants in our study. That said, we were able to detect associations of all-cause mortality with smoking and physical activity, (e.g., 1.9 times higher risk and 40% lower risk, respectively—data not shown).

Strengths of our study included the usage of a nationally representative sample of non-institutionalized, healthy US citizens, assessment of two metrics of usual intake of dietary phosphorus (mg/d and mg/kcal), and fractionation of total dietary phosphorus intake into natural and added. A major strength of our analyses is that the method to calculate added phosphorus in foods is novel, likely more accurate, but needs further validation in future studies. The current study has several limitations. NHANES is an observational trial and is comprised of self-reported intakes which are known to be sensitive to under- and over-reporting of foods [45]. Although several covariate sets were used to remove the impact of variables correlated with phosphorus intake, residual confounding with other factors may exist, especially since added phosphorus is used in only a subset of foods.

Further studies, preferably randomized control trials and longitudinal observational studies, are necessary to fully elucidate the health benefits and risks of total, natural, and added phosphorus intake. The disparate results for natural and added phosphorus intakes within the current study provide support for updating current food composition databases to more accurately account for added phosphorus intake. Similar to the revision of folic acid, vitamin E, and vitamin B12 into separate total, natural, and added intakes and sources, the USDA should consider also applying this strategy to phosphorus.

## Figures and Tables

**Table 1:** Association of total, added, and natural phosphorus individual usual intake ^1,2^ (100 mg/d with Physiological Variables in adults 19 years of age and older, NHANES 1988–1994–2015–2016.

		Added Phosphorus		Natural Phosphorus		Total Phosphorus	
Physiological Variable	*n*	β ± SE	*p*	β ± SE	*p*	β ± SE	*p*
All							
Apolipoprotein B (mg/dL)	12,169	−0.60 (0.59)	0.3138	−0.36 (0.17)	0.0354	−0.42 (0.16)	0.0109
BP diastolic (mean rdg mm hg)	35,481	−0.35 (0.19)	0.0684	−0.25 (0.05)	**<0.0001**	−0.28 (0.05)	**<0.0001**
BP systolic (mean rdg mm hg)	35,643	−0.59 (0.31)	0.0607	−0.32 (0.07)	**<0.0001**	−0.37 (0.07)	**<0.0001**
CVD risk score	33,979	0.004 (0.001)	**0.0004**	−0.001 (0.0003)	**<0.0001**	−0.001 (0.0003)	**0.0008**
Creatinine (mg/dL)	34,856	0.01 (0.004)	**0.0067**	−0.002 (0.001)	0.1156	−0.001 (0.001)	0.4097
GFR (mL/min/1.73 m^2^) (ckd-epi)	34,856	−0.50 (0.26)	0.0588	0.02 (0.08)	0.7831	−0.02 (0.08)	0.8364
Glucose, plasma (mg/dL) *	15,816	0.49 (0.53)	0.3615	0.01 (0.10)	0.9431	0.02 (0.09)	0.8031
Glycohemoglobin (%)	35,302	0.07 (0.01)	**<0.0001**	0.02 (0.003)	**<0.0001**	0.03 (0.003)	**<0.0001**
HDL-cholesterol (mg/dL)	34,952	−2.12 (0.28)	**<0.0001**	0.26 (0.07)	**0.0004**	0.11 (0.07)	0.1336
HOMA-IR	15,724	0.22 (0.11)	0.0408	0.04 (0.03)	0.1254	0.06 (0.03)	0.0250
Insulin (uU/mL) *	15,510	0.03 (0.22)	0.8907	0.01 (0.04)	0.8491	0.01 (0.04)	0.8408
LDL-cholesterol (mg/dL)	15,563	0.15 (0.74)	0.8373	−0.45 (0.24)	0.0632	−0.45 (0.24)	0.0573
Phosphorus (mg/dL)	34,851	0.01 (0.01)	0.3620	0.01 (0.003)	**<0.0001**	0.02 (0.003)	**<0.0001**
Total cholesterol (mg/dL)	34,953	−0.09 (0.71)	0.8953	−0.79 (0.19)	**0.0001**	−0.82 (0.19)	**<0.0001**
Total femur BMC	14,995	0.28 (0.19)	0.1341	0.26 (0.05)	**<0.0001**	0.28 (0.05)	**<0.0001**
Total femur BMD	14,995	0.002 (0.003)	0.5814	0.005 (0.001)	**<0.0001**	0.01 (0.001)	**<0.0001**
Triglyceride (mg/dL) *	15,668	−1.47 (2.22)	0.5084	−0.26 (0.51)	0.6134	−0.28 (0.47)	0.5525
Males							
Apolipoprotein B (mg/dL)	6075	−1.35 (0.72)	0.0635	−0.45 (0.23)	0.0536	−0.57 (0.22)	0.0104
BP diastolic (mean rdg mm hg)	17,956	−0.47 (0.24)	0.0475	−0.29 (0.07)	**<0.0001**	−0.34 (0.07)	**<0.0001**
BP systolic (mean rdg mm hg)	18,030	−0.93 (0.39)	0.0183	−0.33 (0.08)	**0.0001**	−0.41 (0.08)	**<0.0001**
CVD risk score	17,232	0.001 (0.001)	0.2862	−0.0004 (0.0003)	0.2060	−0.0003 (0.0003)	0.3359
Creatinine (mg/dL)	17,603	0.01 (0.005)	0.0582	−0.001 (0.001)	0.6472	0.0001 (0.001)	0.9076
GFR (ml/min/1.73 m^2^) (ckd-epi)	17,603	−0.37 (0.30)	0.2162	−0.07 (0.09)	0.4885	−0.10 (0.10)	0.3207
Glucose, plasma (mg/dL) *	7945	0.91 (0.72)	0.2049	−0.06 (0.12)	0.6259	−0.02 (0.12)	0.8736
Glycohemoglobin:(%)	17,756	0.08 (0.02)	**<0.0001**	0.02 (0.004)	**<0.0001**	0.03 (0.004)	**<0.0001**
HDL-cholesterol (mg/dL)	17,639	−1.65 (0.26)	**<0.0001**	0.09 (0.08)	0.2764	−0.04 (0.09)	0.6642
HOMA-IR	7936	0.35 (0.14)	0.0160	0.06 (0.04)	0.1014	0.09 (0.04)	0.0125
Insulin (uU/mL) *	7837	0.03 (0.30)	0.9180	−0.02 (0.06)	0.7523	−0.01 (0.05)	0.7852
LDL-cholesterol (mg/dL)	7761	−0.48 (0.90)	0.5973	−0.51 (0.33)	0.1233	−0.56 (0.31)	0.0734
Phosphorus (mg/dL)	17,599	0.01 (0.01)	0.5575	0.01 (0.003)	**0.0017**	0.01 (0.003)	**0.0007**
Total cholesterol (mg/dL)	17,639	0.12 (0.94)	0.9015	−1.07 (0.24)	**<0.0001**	−1.09 (0.25)	**<0.0001**
Total femur BMC	7752	0.29 (0.25)	0.2569	0.25 (0.06)	**0.0001**	0.28 (0.06)	**<0.0001**
Total femur BMD	7752	0.002 (0.004)	0.6888	0.004 (0.001)	**0.0013**	0.004 (0.001)	**0.0017**
Triglyceride (mg/dL) *	7893	−3.78 (2.97)	0.2046	−0.35 (0.64)	0.5821	−0.44 (0.60)	0.4637
Females							
Apolipoprotein B (mg/dL)	6094	0.50 (0.90)	0.5820	−0.28 (0.33)	0.3864	−0.26 (0.33)	0.4468
BP diastolic (mean rdg mm hg)	17,525	−0.12 (0.28)	0.6751	−0.15 (0.09)	0.0823	−0.16 (0.08)	0.0541
BP systolic (mean rdg mm hg)	17,613	0.09 (0.41)	0.8282	−0.39 (0.12)	**0.0017**	−0.39 (0.12)	**0.0021**
CVD risk score	16,747	0.01 (0.002)	**<0.0001**	−0.001 (0.0004)	**0.0005**	−0.001 (0.0004)	0.0227
Creatinine (mg/dL)	17,253	0.01 (0.01)	0.0220	−0.003 (0.002)	0.0441	−0.002 (0.002)	0.1551
GFR (ml/min/1.73 m^2^) (ckd-epi)	17,253	−0.77 (0.49)	0.1234	0.21 (0.12)	0.0807	0.16 (0.12)	0.1841
Glucose, plasma (mg/dL) *	7871	−0.34 (0.76)	0.6543	0.16 (0.19)	0.3964	0.13 (0.18)	0.4673
Glycohemoglobin:(%)	17,546	0.05 (0.02)	0.0126	0.02 (0.005)	**<0.0001**	0.03 (0.005)	**<0.0001**
HDL-cholesterol (mg/dL)	17,313	−3.12 (0.60)	**<0.0001**	0.55 (0.13)	**<0.0001**	0.34 (0.12)	**0.0079**
HOMA-IR	7788	0.003 (0.13)	0.9834	0.02 (0.04)	0.5548	0.024 (0.04)	0.5399
Insulin (uU/mL) *	7673	0.02 (0.26)	0.9527	0.09 (0.05)	0.0957	0.08 (0.05)	0.1247
LDL-cholesterol (mg/dL)	7802	0.87 (1.20)	0.4689	−0.45 (0.38)	0.2395	−0.41 (0.41)	0.3201
Phosphorus (mg/dL)	17,252	0.01 (0.02)	0.4739	0.02 (0.004)	**<0.0001**	0.02 (0.004)	**<0.0001**
Total cholesterol (mg/dL)	17,314	−0.94 (0.98)	0.3404	−0.37 (0.33)	0.2536	−0.46 (0.34)	0.1826
Total femur BMC	7243	0.30 (0.22)	0.1889	0.27 (0.06)	**<0.0001**	0.30 (0.06)	**<0.0001**
Total femur BMD	7243	0.004 (0.01)	0.4691	0.01 (0.001)	**<0.0001**	0.008 (0.001)	**<0.0001**
Triglyceride (mg/dL) *	7775	−0.42 (2.68)	0.8755	−0.98 (0.64)	0.1281	−0.89 (0.57)	0.1208

^1^ Results adjusted for age, age ^2^, gender, ethnicity, physical activity level (as vigorous, moderate, and sedentary based on responses to a questionnaire), poverty income ratio (PIR) level (as ratio of household income to government-defined poverty levels separated into three groups: <1.35, 1.35 to ≤1.85, and >1.85), and total caloric intake (as individual usual intake). ^2^ Values are represented as beta coefficient (SE) followed by *p*-value (significance set at <0.01). Beta represents the change in physiological variable per 100 mg change in phosphorus intake. BP (blood pressure), GFR (globular filtration rate), ckd-epi (chronic kidney disease epidemiology collaboration equation), HDL (high-density lipoprotein), HOMA-IR (homeostatic model assessment for insulin resistance), LDL (low-density lipoprotein), BMC (bone mineral content), BMD (bone mineral density). * Variables utilized covariate set above and BMI.

**Table 2:** Odds Ratios for Phosphorus Intake and Physiological Variables ^1,2^ in adults 19+ years of age and older, NHANES 1988–1994–2015–2016.

	Total Sample		Added Phosphorus		Natural Phosphorus		Total Phosphorus	
Physiological Variable	Sample (*n*)	Events (*n*)	OR, 99% CI	*p*	OR, 99% CI	*p*	OR, 99% CI	*p*
All								
BP, elevated	35,481	18,723	0.96 (0.88, 1.05)	0.2615	0.97 (0.94, 1.00)	**0.0053**	0.97 (0.94, 0.99)	**0.0021**
Glucose, elevated *	15,816	7903	0.92 (0.83, 1.02)	0.0365	1.00 (0.98, 1.02)	0.7046	0.99 (0.97, 1.01)	0.4435
HDL, reduced	34,952	15,151	1.21 (1.09, 1.34)	**<0.0001**	0.98 (0.95, 1.00)	0.0203	0.99 (0.97, 1.02)	0.3295
Insulin, elevated *	15,510	5142	1.11 (0.97, 1.28)	0.0479	1.01 (0.99, 1.04)	0.2020	1.01 (0.99, 1.04)	0.1066
LDL, elevated	15,563	11,599	1.01 (0.87, 1.18)	0.8756	0.98 (0.93, 1.02)	0.1606	0.98 (0.93, 1.02)	0.1458
Total cholesterol, elevated	34,953	19,193	1.01 (0.91, 1.12)	0.8132	0.98 (0.95, 1.01)	0.0480	0.98 (0.96, 1.01)	0.0433
Triglycerides, elevated *	15,668	6202	0.95 (0.85, 1.06)	0.2242	0.98 (0.96, 1.01)	0.0696	0.98 (0.96, 1.01)	0.0578
Males								
BP, elevated	17,956	10,038	0.94 (0.85, 1.04)	0.0901	0.95 (0.92, 0.99)	**0.0004**	0.95 (0.92, 0.98)	**0.0001**
Glucose, elevated *	7945	4519	0.95 (0.82, 1.09)	0.2897	1.00 (0.97, 1.02)	0.7648	1.00 (0.97, 1.02)	0.6211
HDL, reduced	17,639	7317	1.14 (1.03, 1.27)	**0.0010**	1.00 (0.97, 1.03)	0.9729	1.01 (0.98, 1.04)	0.3687
Insulin, elevated *	7837	2639	1.15 (0.97, 1.36)	0.0312	0.99 (0.96, 1.02)	0.2805	0.99 (0.97, 1.02)	0.5876
LDL, elevated	7761	5942	0.99 (0.82, 1.20)	0.9051	0.97 (0.92, 1.03)	0.1673	0.97 (0.92, 1.02)	0.1244
Total cholesterol, elevated	17,639	9643	1.00 (0.88, 1.12)	0.9403	0.97 (0.94, 1.01)	0.0497	0.97 (0.94, 1.01)	0.0393
Triglycerides, elevated *	7893	3462	0.93 (0.81, 1.07)	0.1957	0.98 (0.96, 1.01)	0.1445	0.98 (0.96, 1.01)	0.1107
Females								
BP, elevated	17,525	8685	1.03 (0.85, 1.25)	0.6892	0.99 (0.94, 1.05)	0.7374	0.99 (0.94, 1.05)	0.8013
Glucose, elevated *	7871	3384	0.83 (0.68, 1.03)	0.0268	0.98 (0.94, 1.02)	0.2492	0.98 (0.94, 1.02)	0.1181
HDL, reduced	17,313	7834	1.37 (1.16, 1.61)	**<0.0001**	0.94 (0.90, 0.98)	**0.0004**	0.96 (0.92, 1.00)	0.0130
Insulin, elevated *	7673	2503	1.03 (0.81, 1.31)	0.7564	1.07 (1.02, 1.12)	**0.0004**	1.06 (1.01, 1.11)	**0.0007**
LDL, elevated	7802	5657	1.02 (0.80, 1.30)	0.8568	0.98 (0.92, 1.05)	0.5465	0.99 (0.92, 1.06)	0.5676
Total cholesterol, elevated	17,314	9550	1.02 (0.87, 1.20)	0.7503	0.99 (0.94, 1.03)	0.3967	0.99 (0.95, 1.03)	0.4301
Triglycerides, elevated *	7775	2740	0.94 (0.77, 1.14)	0.3920	0.96 (0.92, 1.01)	0.0291	0.96 (0.92, 1.01)	0.0261

^1^ Results adjusted for age, age ^2^, gender, ethnicity, physical activity level (as vigorous, moderate, and sedentary based on responses to a questionnaire), poverty income ratio (PIR) level (as ratio of household income to government-defined poverty levels separated into three groups: <1.35, 1.35 to ≤1.85, and >1.85), and total caloric intake (as individual usual intake). ^2^ Values are represented as odds ratio (lower confidence limit, upper confidence limit) followed by *p*-value (testing hypothesis that odds ratio = 1.0, significance set at <0.01) for 100 mg phosphorus intake. BP (blood pressure) elevated: ≥130 mm Hg systolic BP, or ≥80 diastolic BP, or taking anti-hypertensive medications; elevated fasting glucose: ≥100 mg/dL or taking insulin or other hypoglycemic agents; low HDL-cholesterol: <40 mg/dL in males and <50 mg/dL in females or taking anti-hyperlipidemic medications; elevated fasting insulin: ≥15µU/mL; elevated LDL-cholesterol: ≥100 mg/dL or taking anti-hyperlipidemic medications; elevated total cholesterol: ≥200 mg/dL or taking anti-hyperlipidemic medications; elevated triglycerides: ≥150 mg/dL or taking anti-hyperlipidemic medications. * Variables utilized covariate set above and BMI.

**Table 3:** Phosphorus intake quintile for mortality analyses, in adults 19+ years of age and older, NHANES 1988–1994–2015–2016.

Intake Variable	Quintile 1	Quintile 2	Quintile 3	Quintile 4	Quintile 5
Total Phosphorus (mg)	<1020	1020–1188	1188–1390	1390–1630	>1630
Added Phosphorus (mg)	<140	140–163	163–187	187–230	>230
Natural Phosphorus (mg)	<871	871–1010	1010–1186	1186–1406	>1406
Total Phosphorus Density (mg/kcal)	<0.55	0.55–0.59	0.59–0.61	0.61–0.65	>0.65
Added Phosphorus Density (mg/kcal)	<0.075	0.075–0.083	0.083–0.088	0.088–0.094	>0.094
Natural Phosphorus Density (mg/kcal)	<0.46	0.46–0.50	0.50–0.53	0.53–0.57	>0.57

**Table 4:** Dietary phosphorus intake ^1^ association with mortality by quintile of intake (g) in adults 19+ years of age and older, NHANES 1988–1994.

	Total Sample		Quintile 1		Quintile 2		Quintile 3		Quintile 4		Quintile 5		*p*-Trend ^2^
Phosphorus Intake	Sample (*n*)	Events (*n*)	Events (*n*)	OR, 99% CI	Events (*n*)	OR, 99% CI	Events (*n*)	OR, 99% CI	Events (*n*)	OR, 99% CI	Events (*n*)	OR, 99% CI	
Covariate Set 1 ^3^													
Total	10,481	1425	391	1.00 (ref)	312	0.90 (0.66, 1.22)	324	0.86 (0.54, 1.37)	257	0.64 (0.34, 1.22)	141	0.93 (0.44, 1.96)	0.3587
Added	10,481	1425	593	1.00 (ref)	277	0.88 (0.67, 1.16)	216	0.88 (0.64, 1.20)	215	0.92 (0.63, 1.35)	124	0.74 (0.42, 1.27)	0.2077
Natural	10,481	1425	369	1.00 (ref)	309	0.83 (0.60, 1.15)	322	0.77 (0.51, 1.17)	260	0.54 (0.30, 0.97)	165	0.82 (0.44, 1.52)	0.1134
Covariate Set 2 ^4^													
Total	4014	536	143	1.00 (ref)	117	1.05 (0.68, 1.60)	122	1.21 (0.62, 2.36)	95	1.00 (0.48, 2.05)	59	1.86 (0.79, 4.39)	0.1677
Added	4014	536	224	1.00 (ref)	103	0.75 (0.47, 1.19)	85	0.73 (0.44, 1.22)	83	0.77 (0.45, 1.31)	41	0.63 (0.22, 1.78)	0.1806
Natural	4014	536	128	1.00 (ref)	130	1.02 (0.65, 1.59)	114	1.03 (0.56, 1.88)	96	0.82 (0.41, 1.62)	68	1.58 (0.73, 3.43)	0.3270
Covariate Set 3 ^5^													
Total	4014	536	143	1.00 (ref)	117	1.06 (0.70, 1.62)	122	1.17 (0.60, 2.31)	95	0.98 (0.48, 2.02)	59	1.85 (0.79, 4.37)	0.1851
Added	4014	536	224	1.00 (ref)	103	0.74 (0.46, 1.19)	85	0.74 (0.45, 1.24)	83	0.76 (0.43, 1.35)	41	0.59 (0.19, 1.77)	0.1771
Natural	4014	536	128	1.00 (ref)	130	1.05 (0.66, 1.66)	114	1.01 (0.55, 1.86)	96	0.82 (0.42, 1.61)	68	1.61 (0.74, 3.48)	0.3292

^1^ Data presented as hazard ratio (lower confidence level 99%, upper confidence level 99%). ^2^ P for trend testing if hazard ratio = 1.0 (quintile 1 set as reference and hazard ratio set to 1.0). ^3^ Results adjusted for total/added/natural phosphorus total (g), total/added/natural phosphorus^2^, age, gender, ethnicity, poverty income ratio (PIR) level (as ratio of household income to government-defined poverty levels separated into three groups: <1.35, 1.35 to ≤1.85, and >1.85), Kcal; ^4^ Results adjusted for covariate set 1 and BMI, systolic blood pressure, smoking status (former, current, never), physical activity level (as vigorous, moderate, and sedentary based on responses to a questionnaire), LDL Cholesterol, Log(Albumin Creatinine Ratio), GFR (chronic kidney disease epidemiology collaboration equation), Serum Vitamin D (< 16.2 ng/mL, Serum Vit D ≥ 16.2 ng/mL); ^5^ Results adjusted for covariate set 2 and Healthy Eating Index 2015 and serum Phosphorus (mg/dL).

**Table 5:** Dietary phosphorus intake density ^1^ (mg/kcal) association with mortality by quintile of intake (g).

	Total Sample		Quintile 1		Quintile 2		Quintile 3		Quintile 4		Quintile 5		*p*-Trend ^2^
Phosphorus Intake	Sample (*n*)	Events (*n*)	Events (*n*)	OR, 99% CI	Events (*n*)	OR, 99% CI	Events (*n*)	OR, 99% CI	Events (*n*)	OR, 99% CI	Events (*n*)	OR, 99% CI	
Covariate Set 1 ^3^													
Total	10,481	1425	203	1.00 (ref)	215	0.78 (0.55, 1.11)	241	0.93 (0.62, 1.41)	318	0.87 (0.58, 1.30)	448	0.87 (0.59, 1.26)	0.6451
Added	10,481	1425	503	1.00 (ref)	413	0.87 (0.67, 1.13)	222	0.85 (0.60, 1.21)	139	0.89 (0.58, 1.36)	148	0.90 (0.56, 1.44)	0.4612
Natural	10,481	1425	197	1.00 (ref)	198	1.02 (0.69, 1.50)	215	0.97 (0.65, 1.45)	335	0.98 (0.65, 1.50)	480	1.02 (0.67, 1.54)	0.9353
Covariate Set 2 ^4^													
Total	4014	536	69	1.00 (ref)	83	1.09 (0.57, 2.07)	98	1.61 (0.85, 3.03)	126	1.14 (0.66, 1.99)	160	1.36 (0.76, 2.44)	0.2711
Added	4014	536	195	1.00 (ref)	153	0.74 (0.52, 1.06)	90	0.86 (0.51, 1.47)	52	0.68 (0.33, 1.38)	46	0.88 (0.38, 2.04)	0.4412
Natural	4014	536	67	1.00 (ref)	67	1.04 (0.53, 2.02)	93	1.57 (0.90, 2.75)	137	1.39 (0.76, 2.55)	172	1.54 (0.91, 2.62)	0.0386
Covariate Set 3 ^5^													
Total	4014	536	69	1.00 (ref)	83	1.13 (0.58, 2.19)	98	1.59 (0.85, 2.98)	126	1.17 (0.67, 2.04)	160	1.40 (0.77, 2.56)	0.2348
Added	4014	536	195	1.00 (ref)	153	0.74 (0.52, 1.06)	90	0.85 (0.49, 1.48)	52	0.68 (0.32, 1.43)	46	0.86 (0.36, 2.07)	0.4550
Natural	4014	536	67	1.00 (ref)	67	1.09 (0.56, 2.10)	93	1.59 (0.90, 2.82)	137	1.43 (0.78, 2.60)	172	1.62 (0.91, 2.86)	0.0394

^1^ Data presented as hazard ratio (lower confidence level 99%, upper confidence level 99%). ^2^ P for trend testing if hazard ratio = 1.0 (quintile 1 set as reference and hazard ratio set to 1.0). ^3^ Results adjusted for total/added/natural phosphorus total (g), total/added/natural phosphorus^2^, age, gender, ethnicity, poverty income ratio (PIR) level (as ratio of household income to government-defined poverty levels separated into three groups: <1.35, 1.35 to ≤1.85, and >1.85), Kcal; ^4^ Results adjusted for covariate set 1 and BMI, systolic blood pressure, smoking status (former, current, never), physical activity level (as vigorous, moderate, and sedentary based on responses to a questionnaire), LDL Cholesterol, Log(Albumin Creatinine Ratio), GFR (chronic kidney disease epidemiology collaboration equation), Serum Vitamin D (< 16.2 ng/mL, Serum Vit D ≥ 16.2 ng/mL); ^5^ Results adjusted for covariate set 2 and Healthy Eating Index 2015 and serum Phosphorus (mg/dL).

## Data Availability

The data presented in this study are from publicly available data in NHANES and other additional data are available in the article and Supplementary Material.

## Supplementary Materials

The following supporting information can be downloaded at: https://www.mdpi.com/article/10.3390/nu14091738/s1, Table S1: Exclusions for mortality analyses.
