# Supplementary material for: Association of Total, Added, and Natural Phosphorus Intakes with Biomarkers of Health Status and Mortality in Healthy Adults in the United States
Source: Nutrients. 2022 Apr 22;14(9):1738. doi: 10.3390/nu14091738 (PMC9104875; doi:10.3390/nu14091738)
Supplement: Supplementary file 1 [file nutrients-14-01738-s001.zip › nutrients-1662408-supplementary.pdf]

nutrients
MDPI

Supplementary Materials

Association of Total, Added, and Natural Phosphorus Intakes with Biomarkers of Health Status and Mortality in Healthy Adults in the United States

Kristin Fulgoni [1], Victor L. Fulgoni III [1,*] and Taylor C. Wallace [2,3]

Table S1. Exclusions for mortality analyses.

Variable	Variable Exclusions	Total Exclusions	Inclusions
20<= age <= 80			15,020
Pregnant	282	282	14,738
Lactating	91	368	14,652
Smoking status missing	1	369	14,651
Physical Activity Level Missing	264	629	14,391
Poverty Income Ratio Level Missing	1,349	1,908	13,112
Antidiabetic Medication Status Missing	0	1,908	13,112
Told had Diabetes	1,205	2,940	12,080
Taking Antidiabetic Medication	773	2,952	12,068
Told had Acute Myocardial Infarction	595	3,355	11,665
Told had Congestive Heart Failure	496	3,498	11,522
Told had Stroke	346	3,648	11,372
Told had Cancer	930	4,291	10,729
Kcal < 1st Percentile	149	4,440	10,580
Kcal > 99th Percentile	99	4,539	10,481
